# Candidemia in an Orthopedic Patient Detected Coincidentally by Peripheral Blood Smear

**DOI:** 10.3390/diagnostics14222597

**Published:** 2024-11-19

**Authors:** Eirini Spatha, Loredana-Mariana Gheorghe, Ioulia Chaliori, Nikolaos J. Tsagarakis, Nikolaos Patsiogiannis, Sofia K. Chaniotaki

**Affiliations:** 1 Haematology Laboratory, Peripheral General Hospital of Athens Georgios Gennimatas, 11527 Athens, Greece; eirini.spatha@outlook.com (E.S.); nikolaostsagarakis@gmail.com (N.J.T.); 2 First Orthopaedic Department, Peripheral General Hospital of Athens Georgios Gennimatas, 11527 Athens, Greece; nickpats@gmail.com

**Keywords:** candidemia, bloodstream infection, periprosthetic joint infection, phagocytosed blastospores, pseudohyphae, monocytes, neutrophils, peripheral blood smear, multiplex PCR

## Abstract

An elderly male, with a recent COVID-19 infection and cardiovascular comorbidities, experienced a prolonged hospitalization due to a periprosthetic joint infection (PJI) and bacteremia, post hip hemiarthroplasty. Despite the initial clinical improvement while on targeted antimicrobial therapy, the patient later developed a low-grade fever and signs of myelosuppression. In the May–Grünwald–Giemsa stain of peripheral blood smear (PBS), pseudohyphae among red blood cells (RBCs) and phagocytosed blastospores in neutrophils and monocytes were detected, indicating candidemia rather than contamination of the stain. Echinocandin treatment was immediately initiated, and *Candida albicans* was identified from the blood culture, using multiplex polymerase chain reaction (PCR). Despite the early initiation of antifungal therapy and the removal of the central venous line (CVL), the patient passed away within 24 h. Candidemia is a leading cause of nosocomial bloodstream infections with high morbidity and mortality and is associated with multiple risk factors including surgery, CVLs, prolonged hospitalization, concomitant bacterial infection, broad-spectrum antibiotics, and immunosuppression. Isolation from blood cultures remains the gold standard for diagnosing candidemia. Detection of candidemia by PBS is extremely rare, requires an experienced microscopist, and is considered to be an emergency. Clinical suspicion, early laboratory identification, and immediate clinician notification are crucial for prompt antifungal treatment.

**Figure 1:**
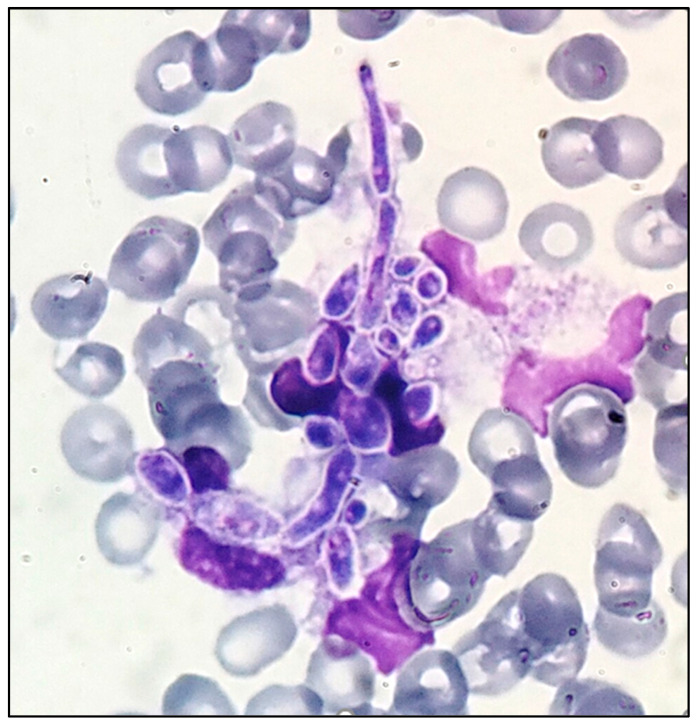
Peripheral blood smear with yeast’s blastospores and pseudohyphae among red blood cells (RBCs) (May–Grünwald–Giemsa stain (MGG); original magnification, ×1000). Invasive candidiasis includes both candidemia and deep-seated tissue candidiasis and typically affects hospitalized patients. It has been reported to impact more than 250,000 people globally, causing more than 50,000 deaths annually. Candidemia is the most common manifestation of invasive candidiasis and is listed in the literature as the fourth most common bloodstream infection and a leading cause of nosocomial bloodstream infections, with very high morbidity and mortality (as high as 40%, even when patients receive antifungal therapy) [1,2]. Detection of candidemia by peripheral blood smear (PBS) is rare, requiring high yeast concentration (typically ≥ 1–5 × 10^5^ colony-forming units (CFU)/mL) and evaluation by an experienced microscopist [2,3,4]. It constitutes an emergency, indicating advanced infection and immediate treatment is required [2]. Finding phagocytosed blastospores in the cytoplasm of leukocytes, along with free blastospores and pseudohyphae, is crucial to rule out contamination of the stain and confirm an actual candidemia case [4]. Surgical Site Infection (SSI) is a major complication following hip or knee prosthesis, with early-onset infection occurring within the first three months after surgery. The incidence of periprosthetic joint infection (PJI) following primary total hip arthroplasty (THA) is lower than in knee arthroplasty, estimated at approximately 1%. The vast majority of PJIs are bacterial, while fungal prosthetic joint infections (fPJIs) constitute about 1–2% of all PJIs and are commonly caused by *Candida* spp. A total of 372 cases of fPJI have been reported in the literature, with 161 of these being complications of THA [5]. An elderly male, with a history of coronary artery bypass graft (CABG) and hypertension, was hospitalized in the Orthopedic Clinic due to a PJI and bacteremia following hip hemiarthroplasty. The patient was initially admitted for a subcapital femoral fracture resulting from a fall from standing height, while there was also a history of a recent hospitalization due to SARS-CoV-2 infection, treated with remdesivir and dexamethasone. He was discharged postoperatively with instructions for mobilization and wound cleansing; however, after a follow-up, the presence of periprosthetic infection was established. The initially performed surgical debridement of the arthroplasty failed to control the infection. Subsequently, the patient underwent the removal of the prosthesis (Girdlestone procedure) and VAC (vacuum-assisted closure) was also used to promote wound healing. Transfusions and fluid/electrolyte replenishment were also required. The placement of a central venous line (CVL), for administration of targeted antibiotic therapy against isolated pathogens, was deemed necessary. The isolated microorganism from blood cultures was methicillin-resistant *S. aureus* (MRSA) and from wound cultures (tissue, pus) MRSA, *P. aeruginosa*, and *P. mirabilis*. Initially, the patient showed improvement and became hemodynamically stable, after recovering from MRSA bacteremia. The blood cultures were negative and the complete blood count (CBC) was normal, so there was no reason to examine a PBS with May–Grünwald–Giemsa stain (MGG) at that time. However, over time, and while on targeted antibiotics for the PJI, the CBC revealed signs of myelosuppression. Low-grade fever occurred, and the latest CBC showed severe thrombocytopenia. Urine analysis showed blastospores, and a PBS was prepared to confirm the findings of the CBC. In the MGG of the PBS, pseudohyphae and free blastospores among red blood cells (RBCs) were coincidentally detected (Figure 1).

**Figure 2:**
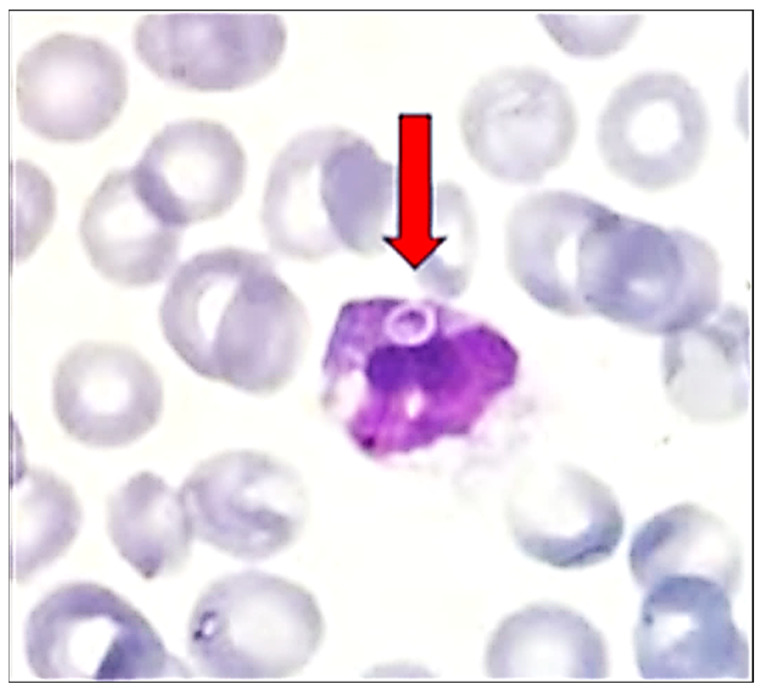
Peripheral blood smear with yeast’s blastospore in the cytoplasm of a monocyte (red arrow): the first blastospore that was detected in a white blood cell (WBC) (MGG; original magnification, ×1000). Further assessment of the smear confirmed our initial findings; a phagocytosed blastospore was detected in the cytoplasm of a monocyte, marking the first blastospore that was detected in a white blood cell (WBC) (Figure 2). Candidemia is associated with multiple risk factors [6,7] and communication with the attending orthopedic surgeon regarding the patient’s history raised suspicion, confirming the presence of many of them: previous surgery, CVL and Foley placement, prolonged hospitalization, concomitant bacterial infection, and broad-spectrum antibiotics use.

**Figure 3:**
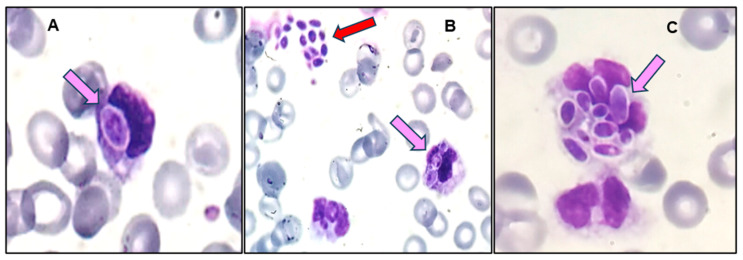
Peripheral blood smear with yeast’s blastospores in the cytoplasm of a monocyte (**A**) and a neutrophil (**B**,**C**): pink arrows and free among RBCs (**B**) red arrow; (MGG; original magnification, ×1000). Meticulous examination of the blood smear further revealed phagocytosed blastospores in both monocytes and neutrophils, as well as another site of free blastospores among RBCs (Figure 3). The patient showed no signs of respiratory distress, had an adequate gas exchange, and therefore was not in the intensive care unit (ICU) but in the wards. Empirical antibiotic treatment is initiated in every patient with a suspected blood infection after obtaining blood cultures. Prophylactic antifungal therapy is not routinely administered and is more commonly reserved for immunocompromised patients. Antifungal therapy is immediately initiated after notification by the Microbiology Laboratory. The primary characteristic that prompted consideration of fungal infection was the presence of pseudohyphae and phagocytosed blastospores in the PBS. Empirical treatment was initiated after communication with the attending physician about the observed fungus in the PBS. An echinocandin was administered for suspected candidiasis, following the guidelines [8,9]. The patient received anidulafungin, on a loading dose of 200 mg × 1 on day 1, followed by a daily maintenance dose of 100 mg × 1. Due to the emergence of non-albicans *Candida* spp. and a history of previous hospitalizations, increasing the risk of possible exposure to *C. auris*, the use of an azole was avoided. Blood cultures were immediately obtained, following communication, and were urgently processed using BIOFIRE FILMARRAY TORCH System (BioMérieux, Marcy-l’Étoile, France) multiplex polymerase chain reaction (PCR), by the Microbiology Laboratory. Typically, *Candida* is detected, by microbiologists, in the Gram stain of positive blood cultures, and prompt notification of the clinician is required to start empirical antifungal treatment. Isolation from positive blood cultures remains the gold standard for diagnosing candidemia [7]. Positive blood cultures are routinely inoculated on blood agar, which is a non-selective medium (growth of Gram-positive, Gram-negative bacteria, fungi), and a MacConkey No.2 agar, which is a differential medium (growth of Gram-negative and enterococci). In case blastospores are observed in the gram stain, a sabouraud dextrose agar is added as a selective medium for fungi. A selective and differential chromoagar can be further used for a rapid, primary identification of the fungal subspecies. Identification and antifungal susceptibility testing, using Vitek2 (BioMérieux, Marcy-l’Étoile, France) and Kirby-Bauer disc diffusion method, showed fluconazole-sensitive *C. albicans* in both the urine and the blood culture of the patient. On the day of the diagnosis, low-grade fever and an elevated c-reactive protein (CRP) value were marked while the patient was on antibiotic therapy. Blastospores in the urine analysis were observed one day prior to the diagnosis and a urine culture was performed, identifying fluconazole-sensitive *Candida albicans*. It should be noted that CRP levels never normalized after the PJI; however, further elevation of CRP was noted on the day after the diagnosis. Despite the echinocandin administration (two doses) and the CVL removal, the patient succumbed within 24 h of the initial diagnosis. Among patients with candidemia, overall 90-day mortality was estimated at 43% [8]. The *Candida* concentration in the blood of patients with candidemia is usually low (~10 CFU/mL), and the detection of candidemia by using PBS examination requires a yeast concentration of at least 1 × 10^5^ CFU/mL. Therefore, the rare finding of yeast-like fungi (*Candida* species) in peripheral blood smear requires an experienced microscopist and suggests extremely progressive fungemia, which is a life-threatening emergency [2]. Early laboratory identification and clinician notification can lead to effective antifungal treatment and reduce mortality. Despite the unfortunate outcome, the necessity of interdisciplinary collaboration to improve patient care was underlined. We would like to alert every laboratory doctor and emphasize the importance of microscopic findings as a keystone to prompt diagnosis and treatment of clinical entities with non-specific initial manifestations.

## Data Availability

Data sharing is not applicable to this article as no datasets were generated or analyzed during the current study.
